# Aortic flow changes during free breathing exercise, measured with real time MRI

**DOI:** 10.1186/1532-429X-18-S1-P324

**Published:** 2016-01-27

**Authors:** Gergely V Szantho, Tamas Erdei, Chris B Lawton, Mark Hamilton

**Affiliations:** 1 CMR, Bristol Heart Institute, Bristol, United Kingdom; 2 Cardiology, University Hospitals Wales, Cardiff, United Kingdom

## Background

Measuring flow with MRI is well established. It is challenging, however, to detect beat-to-beat blood flow response to exercise. We used a new real-time sequence to measure aortic flow during exercise. This phase contrast echo planar imaging (EPI) sequence was previously validated against flow phantoms, and we have recently validated it against the clinical standard sequence in vivo at rest.

We set out to establish a methodology of measuring ascending aortic flow during free breathing exercise.

## Methods

A non-gated phase contrast EPI sequence (WiP#720 Siemens, Erlangen, Germany) was used to measure ascending aortic flow in 12 healthy volunteers at rest and during two stages of free breathing exercise in a 1.5T Magnetom Avanto MRI scanner. Real time EPI parameters: temporal resolution 38.72 ms, echo time 2.79 ms, EPI factor 7, typical voxel size ≍2.5 × 3.2 × 10 mm, flip angle 30°; phases: 258 (≍10s) at rest and 155 (≍6s) on exercise in order to include a full breathing cycle in each acquisition. Beat-to-beat analysis was performed in Argus, Siemens software with manually contouring the ascending aorta in each phase.

An MRI-compatible, supine, pedal ergometer was used to achieve steady heart rates (HR) of 150% and 180% of resting.

We expressed stroke volume (SV) as the mean of the beat-to-beat values of the actual acquisition. Net flow was calculated from the SV multiplied by the HR. Peak velocity (PV) and peak flow rate (PFR) values were taken from the cardiac cycle with the largest SV of the actual acquisition. Mean values of all volunteers' measurements and their standard deviation are used for data presentation. Student's paired t-test is used to show the significance of differences.

Flow datasets of five volunteers were independently re-analysed for intra-observer variability of SV by one observer and for inter-observer variability of SV by two independent observers. Intra- and inter-observer variability was assessed by a two-way mixed effects model intra-class correlation coefficient (ICC).

## Results

All acquired images were evaluable. We recorded a physiological response to two levels of exercise as follows: Net flow (l/min) 5.63 ± 1.11 vs 8.84 ± 1.51 vs 11.39 ± 1.12 (p < 0.0001 and p < 0.0002 respectively); PFR (ml/s) 486.59 ± 82.43 vs 601.57 ± 177.53 vs 723.44 ± 186.24 (p < 0.02 and p = 0.08 NS respectively).

Differences between PV and SV values were only significant between rest and the higher level of exercise: PV (cm/s) 110.15 ± 12.58 vs 131.55 ± 26.51 (p < 0.01); SV (ml) 98.24 ± 19.92 vs 106.27 ± 22.29 (p < 0.05), the latter showing that the increase in net flow was primarily driven by the change in HR.

The intra-observer variability of SV was excellent both at rest (ICC = 0.985) and on exercise (ICC = 0.983). The inter-observer variability of SV showed similarly good agreement at rest (ICC = 0.975) and also on exercise (ICC = 0.962).

## Conclusions

The physiological changes in aortic flow can be measured with EPI phase contrast MRI during exercise, which gives rise to further studies of exercise-physiology and pathology.

